# The willingness of parents to vaccinate their children younger than 12 years against COVID-19: a cross-sectional study in Malaysia

**DOI:** 10.1186/s12889-022-13682-z

**Published:** 2022-06-29

**Authors:** Diana-Leh-Ching Ng, Gin-Gin Gan, Chee-Shee Chai, Nur Adila Bt Anuar, Woweham Sindeh, Wei-Jing Chua, Asri B. Said, Seng-Beng Tan

**Affiliations:** 1 Department of Medicine, Faculty of Medicine and Health Science, University Malaysia Sarawak, Kota Samarahan, Sarawak Malaysia; 2 Department of Medicine, Faculty of Medicine, University of Malaya, Kuala Lumpur, Malaysia; 3 Department of Primary Care Medicine, Faculty of Medicine, University of Malaya, Kuala Lumpur, Malaysia

**Keywords:** COVID-19 vaccine, Children, Vaccine acceptance, Vaccine hesitancy, Vaccine refusal

## Abstract

**Background:**

The initiation of a new drug, for instance, the coronavirus disease 2019 (COVID-19) vaccine in children could be a source of major concern for parents. This study aims to determine the willingness of parents in Malaysia to vaccinate their children younger than 12 years against COVID-19.

**Methods:**

An online cross-sectional survey was conducted nationwide in Malaysia from August 29, 2021, to October 17, 2021. Parents with children younger than 12 years were enrolled via the snowball sampling method.

**Results:**

The analysis included data from 3,528 parents (79.5%) of the 4,438 survey responses received. Of these parents, 2,598 (73.6%) were willing, 486 (13.8%) were not willing, and 444 (12.6%) were still hesitant to vaccinate their children against COVID-19. Single parents (odds ratio [OR], 2.0; 95% confidence interval [CI], 1.32–3.04; *P* = 0.001), parents with secondary or lower education (OR, 1.5; 95% CI, 1.21–1.96; *P* < 0.001), healthcare workers (OR, 1.7; 95% CI, 1.34–2.26; *P* < 0.001), parents who had significant contact with COVID-19 (OR, 1.3; 95% CI, 1.09–1.63; *P* = 0.006), and parents who had been vaccinated against COVID-19 (OR, 15.4; 95% CI, 9.76–24.33; *P* < 0.001) were found more willing to immunize their children. The common reasons for vaccination given by parents who were willing to immunize their children include protection of children (99.4%), protection of other family members (99.3%), and effectiveness (98.2%). The common reasons against vaccination given by parents who were not willing to immunize their children were uncertainty about the new vaccine (96.1%), concerns about vaccine contents (93.2%), limited vaccine information from physicians (82.3%), and the belief of vaccine was unsafe (79.8%).

**Conclusions:**

In this study, nearly three-quarters of parents were willing to vaccinate their children younger than 12 years against COVID-19. The parents’ history of COVID-19 vaccination was the strongest independent predictor of their willingness to vaccinate their children. Therefore, future health education for the COVID-19 vaccine should focus on parents who are prone to vaccine refusal or hesitation, address the common reasons for vaccine refusal, and highlight the vaccine’s benefits.

**Supplementary Information:**

The online version contains supplementary material available at 10.1186/s12889-022-13682-z.

## Background

Immunization is the most reliable and cost effective public health intervention that has ever been implemented in human history, with millions of lives saved each year [1, 2]. A vaccine confers protection against pathogens via two mechanisms [3]. First, it stimulates B cells to produce neutralizing antibodies that provide immediate immunity [4]. Second, it induces the production of antigen-specific memory T cells that persists even long after the pathogens have been eliminated resulting in enhanced immune response in case of a subsequent infection [5]. To date, immunization programs have successfully controlled several contagious diseases worldwide, such as smallpox, polio, diphtheria, pertussis, and rubella [6].

Since its first emergence in December 2019, coronavirus disease 2019 (COVID-19) has spread rapidly to every part of the world, and as it became a global public health emergency, it was declared a pandemic. As of March 10, 2022, a total of 452 million people have been infected with COVID-19 worldwide, resulting in six million deaths [7]. Although the control of human movement, including lockdown, travel restriction, and quarantine, is an effective containment and mitigation strategy for COVID-19, it could lead to significant mental health problems [8] and socioeconomic burden [9]. Pandemic fatigue, defined by the World Health Organization (WHO). as a demotivation to follow recommendations, which is a consequence of prolonged public health measures and restrictions, may contribute to the rebound of COVID-19 cases [10].

The rapid development of a vaccine against COVID-19 offers hope for everyone to see an end to the pandemic. The WHO has issued an emergency use listing for nine COVID-19 vaccines for adults as of January 12, 2022 [11]. Meanwhile, the US Food and Drug Administration granted the emergency use of the Pfizer-BioNTech COVID-19 vaccine in teenagers aged 12 years and older on May 10, 2021, and in children aged 5–11 years on October 29, 2021, respectively. Phase 3 studies reported these vaccines to be efficacious in terms of a reduction in the numbers of symptomatic infections, hospitalizations, critical illness, and deaths, as well as safety profile. As of March 10, 2022, at least one dose of the COVID-19 vaccine has been administered to 63.4% of the world population, which translates into a total of 10.93 billion doses administered globally [12].

In Malaysia, the Crisis Preparedness and Response Center of the Ministry of Health recently highlighted that 15.3% of COVID-19 cases were detected among children. By August 30, 2021, an alarming number of 310,074 children had been diagnosed with COVID-19, leading to 41 deaths. Even though children with COVID-19 tend to be asymptomatic and have a lower mortality rate [13], they can still experience other complications, such as mental stress during isolation, social stigmatization, and long-term lung complications. Moreover, multisystem inflammatory syndrome in children is a rare but potentially fatal complication of the disease [14].

Vaccination in children often requires the consent of parents or guardians [15], and the administration of a new agent, such as a COVID-19 vaccine, in children could be a source of major concern because of various factors. The first concern is regarding what is the most appropriate dosage of COVID-19 vaccine for children of different age groups? Studies have shown children often require a different dosage of vaccine from adults and even among themselves because of distinct pharmacokinetics that is influenced by age, body composition, liver and kidney function, and enzyme system maturity [16]. The second concern is how to monitor the short-term side effects of the vaccine among the children? Children, especially those at a younger age, may have difficulty to understand the monitoring instruction and expressing their problems. Moreover, some complications such as myocarditis and pericarditis were reported more commonly in the younger and were difficult to detect [17]. The third concern is the unforeseen long-term side effects of the vaccine. Children are more prone to experience potential long-term adverse effects because the vaccine is given at a much younger age. These potential adverse effects include autoimmune disease, infertility, and tumour.

By August 29, 2021, Malaysia recorded a total of 1.7 million cases of COVID-19, resulting in 16 087 deaths [7]. The country shifted from the movement control phase to a recovery phase following a high vaccination rate against COVID-19 among adults. The administration of COVID-19 vaccines to teenagers aged 12 years and older commenced on September 15, 2021, whereas that for children younger than 12 years was only started on February 3, 2022. -. This study aimed to assess the willingness of parents in Malaysia to vaccinate their children younger than 12 years against COVID-19. Furthermore, the sociodemographic and clinical factors that influenced parents’ willingness to vaccinate their children were also identified.

## Methods

### Study design and patients

We conducted a nationwide online cross-sectional study in Malaysia from August 29, 2021, to October 17, 2021. The inclusion criteria of the study were as follows: participants 18 years of age and above, parent of at least one child younger than 12 years, and currently resident in Malaysia. Participants who were unable to read English, Malay, or Mandarin, and those not given consent were excluded from the study. The sample size was calculated based on a formula for cross-sectional study [sample size = *Z*_1-α_^2^ p(1 − p)/*d*^2^] [18], in which *Z* was the confidence interval at 95%, *d* was the margin of error of 5%, and p was the proportion of parents willing to allow their children to receive the COVID-19 vaccine at 48.2% based on a recent study [19]. Therefore, the minimum sample size for this study was 665 participants.

This study was conducted according to the Declaration of Helsinki. Online informed written consent was obtained from every respondent before the commencement of the study. Ethics approval was obtained from the Medical Research and Ethics Committee of the University Malaya Medical Center (MECID. No: 202182–10,437).

### Procedure

An online survey was used for the data collection because of the ongoing COVID-19 pandemic in the country. The respondents were recruited using the snowball sampling method. First, the researchers shared the survey with their contacts that fit the study criteria and invited them to become the respondents. Next, these agreeable respondents were asked to share the survey with their contacts who also fit the study criteria and potentially willing to be the next respondents. Similar recruitment process was repeated until adequate numbers of respondents were obtained. In this survey, each respondent received an advertisement and a questionnaire in Google Forms via mobile messaging service on WhatsApp. The respondents were asked to complete an online consent form after confirming that they understood the purpose, risks, and benefits of the study. This anonymous self-administered questionnaire was available in three languages: English, Malay, or Mandarin. Time for completion of questionnaire was approximately 10–15 min. No incentive was offered for completing the questionnaire.

The questionnaire was developed by a group of COVID-19 immunization experts who reviewed the available literature and discussed it, taking into account the local situation and policies [20–24]. The face validity of the questionnaire was assessed through a pilot study of 100 lay people, and the content validity was assessed by a group of clinicians with vast experience in patient surveys. The survey consisted of three parts: 1) the sociodemographic characteristics and clinical data of parents, 2) the demographic characteristics and clinical data of children, and 3) the willingness of the parents to allow their children to get the COVID-19 vaccine, and reasons for the decision. The sociodemographic characteristics of the parents included age, gender, marital status, region of residence, education level, household income, employment status, number of their children that younger than 12 years, whether the respondents were healthcare workers, and whether they lived with an elderly person or someone with a chronic illness. The clinical data of the parents included information on exposure to someone with COVID-19; whether they had been diagnosed with COVID-19; whether their families, relatives, or colleagues had been diagnosed with COVID-19; and their COVID-19 vaccination status. The demographic characteristics of the children included age, and clinical data included information on chronic diseases. Survey response options concerning parents’ willingness to vaccinate their children against COVID-19 included “Yes,” “No,” and “Unsure.” This was followed by a list of reasons for their decision, and the available responses were “Yes” and “No,” except for “other reasons” for which a written answer was required. The regions of residence were as follows: the Central region (Selangor, Kuala Lumpur, and Putrajaya), the Southern region (Johor, Melaka, and Negeri Sembilan), the Northern region (Perak, Penang, Kedah, and Perlis), East Coast (Pahang, Kelantan, and Terengganu), and Borneo (Sabah, Sarawak, and Labuan). Income classification of the population in Malaysia was calculated on the basis of the household income as follows: the B40 group which is the bottom 40% (less than RM 6200 per month); M40, the middle 40% (RM 6200–RM 13,000 per month); and T20, the top 20% (more than RM 13,000 per month) [25]. Significant contact with someone who had COVID-19 was further divided into that which required quarantine and that which required self-surveillance. Children were divided into three age groups: 5–11 years, 2–4 years, and below 2 years. This division was based on the development and dosage of COVID-19 vaccines in clinical trials.

Parents who were willing to vaccinate their children against COVID-19 had 13 reasons listed to choose from (Supplement Fig. 1). Two reasons regarded vaccine properties; three, the recommendation of the vaccine; three, the severity of COVID-19 in the country; two, targets protected by the vaccine; and three, freedom after vaccination.

Parents who were not willing to vaccinate their children against COVID-19 had 16 reasons listed to choose from. Four reasons regarded vaccine properties; three, limited information about the vaccine; five, characteristics of the children; three, postvaccination freedom being not of importance; and one, cultural and religious concerns.

Parents who were still hesitant about vaccinating their children against COVID-19 had 13 factors that could affect their decision in the future listed to choose from. Four reasons regarded vaccine properties; three, recommendations; two, the severity of COVID-19 in the country at the time of survey completion; three, freedom after vaccination; and one, outcomes of the vaccination program in other countries. Hesitancy to a vaccine was defined as a continuum between full acceptance and outright refusal of that vaccine [26].

### Statistical analysis

Categorical variables were expressed as percentages, whereas continuous variables were expressed as mean (SD) or median with interquartile range. Differences in variables were examined between parents who were willing to vaccinate their children against COVID-19 versus those who were not willing or hesitant. Differences in categorical variables were compared using the chi-square test, whereas differences in continuous variables were compared using an independent *t* test. Gender of parents [27]. household income [28], and variables that significantly differed in the between-group comparison were included as independent variables of the multivariate analysis using logistic regression to predict parents who were willing to vaccinate their children against COVID-19. A two-tailed *P* value of less than 0.05 was considered significant. Statistical analyses were conducted using the SPSS software (SPSS for Windows version 25.0, SPSS Inc, Chicago, IL, USA).

## Results

### Sociodemographic characteristics and clinical data of the parents and their children

Of the 4,438 survey responses received, 3,965 respondents (89.3%) agreed to participate in the study. After excluding 437 (9.8%) respondents who did not have children younger than 12 years, the final sample included 3,528 parents (79.5%). Table 1 shows the sociodemographic characteristics and clinical data of these parents and their children.

**Table 1:** Sociodemographic characteristics and clinical data of the parents and their children

Parameters	Total respondents, *n* = 3,528
**Age, (mean ± SD, 95% CI)** Years	38.6 ± 6.64, 38.37 – 38.83
**Gender, n (%)**	
Mother	2,495 (70.7)
Father	1,033 (29.3)
**Marital status, n (%)**	
Married couple	3,337 (94.6)
Single parent	191 (5.4)
**Region, n (%)**	
Central	2,057 (58.3)
Southern	488 (13.8)
Northern	325 (9.2)
East coast	125 (3.5)
Borneo	533 (15.1)
**Education level, n (%)**	
Tertiary	2,951 (83.6)
Secondary and below	577 (16.4)
**Household income, n (%)**	
B40	1,201 (34.0)
M40	1,517 (43.0)
T20	810 (23.0)
**Employment status, n (%)**	
Employed	2,793 (79.2)
Unemployed	700 (19.8)
Retired	35 (1.0)
**Healthcare workers, n (%)**	
No	3,006 (85.2)
Yes	522 (14.8)
**Lived with elderly or someone with chronic illness, n (%)**	
No	2,576 (73.0)
Yes	952 (27.0)
**History of contact with COVID-19, n (%)**	
No	2,668 (75.6)
Yes	860 (24.4)
*Required quarantine*	*344 (9.8)*
*Required self-surveillance*	*516 (14.6)*
**Had been diagnosed with COVID-19, n (%)**	
No	3,297 (93.5)
Yes	198 (5.6)
Prefers not to answer	33 (0.9)
**Families, relatives, colleagues diagnosed with COVID-19, n (%)**	
No	1,140 (32.3)
Yes	2,388 (67.7)
**Vaccinated for COVID-19, n (%)**	
No	130 (3.7)
Yes	3,332 (94.4)
Prefers not to answer	66 (1.9)
**Children under 12 years, (median, IQR)**	
Number	2, 1 – 3
**Age groups of children, n (%)**	
5 – 11 years	2,703 (76.6)^a^
2 – 4 years	1,691 (47.9)^a^
Below 2 years	567 (16.1)^a^
**Children had chronic illness, n (%)**	
No	3,345 (94.8)
Yes	132 (3.7)
Prefers not to answer	51 (1.4)

^a^from the total of 3,528 respondents

Italic: subgroup of parents with COVID-19 contact

*SD* Standard deviation, *CI* Confidence interval, *COVID-19* Coronavirus 2019, *B40*, lowest 40% of family income group, *M40* Middle 40% of family income group, *T20* Top 20% of the family income group, *IQR* Interquartile range

The mean (SD) age of the parents was 38.6 (6.64) years. The majority of the respondents were mothers (70.7%), married couples (94.6%), from the Central region of Malaysia (58.3%), currently working (79.2%), non-healthcare workers (85.2%) and had tertiary education (83.6%). The family income distribution of the study participants was not much different from that observed for the whole country: 34.0%, 43.0%, and 23.0% belonged to B40, M40, and T20 groups, respectively.

A total of 27.0% of respondents were staying with an elderly person or someone with a chronic disease, and 24.4% of respondents had a history of significant contact with someone who had COVID-19, of which 9.8% required quarantine and 14.6% required self-surveillance. Only 5.6% of the parents had been diagnosed with COVID-19, and 0.9% refused to disclose the information. Most of the parents had members of the family, relatives, or colleagues that had been diagnosed with COVID-19 (67.7%). A vast majority of them (94.4%) had received the COVID-19 vaccine.

The median (interquartile range) number of children younger than 12 years per parent was 2 (1–3). Children of 76.6% of parents were aged 5–11 years; 47.9%, 2–4 years; and 16.1%, below 2 years. Children of 3.7% of the parents had a chronic disease, and 1.4% of the respondents did not disclose the information.

### The willingness of the parents to vaccinate their children against COVID-19 and factors that influenced their decision

Of the 3,528 parents, 2,598 (73.6%) were willing to vaccinate their children against COVID-19, whereas the remaining 486 (13.8%) were not willing to do so, and 444 (12.6%) were hesitant (Table 2).

**Table 2:** The willingness of the parents to vaccinate their children against COVID-19

Parameters	Willing, *n* = 2,598 (73.6%)	Not willing or unsure, *n* = 930 (26.4%)	*P* value*	Multivariate analysis, OR (95% CI), *P* value^#^
**Age, (mean ± SD, 95% CI)** Years	38.5 ± 6.67, 38.18 – 38.73	39.0 ± 6.56, 38.55 – 39.44	0.046	1.0 (0.99 – 1.00), 0.257
**Gender, n (%)**				
Mother	1,831 (70.5)	664 (71.4)	0.597	Ref
Father	767 (29.5)	266 (28.6)		1.0 (0.87 – 1.25), 0.652
**Marital status, n (%)**				
Married couple	2,441 (94.0)	896 (96.3)	0.006	Ref
Single parent	157 (6.0)	34 (3.7)		2.0 (1.31 – 3.04), 0.001
**Region, n (%)**				
Central	1,525 (58.7)	532 (57.2)	0.001	Ref
Southern	365 (14.0)	123 (13.2)		1.1 (0.84 – 1.38), 0.584
Northern	210 (8.1)	115 (12.4)		0.7 (0.53 – 0.91), 0.008
East coast	86 (3.3)	39 (4.2)		0.8 (0.51 – 1.18), 0.226
Borneo	412 (15.9)	121 (13.0)		1.2 (0.96 – 1.59), 0.103
**Education level, n (%)**				
Tertiary	2,139 (82.3)	812 (87.3)	< 0.001	Ref
Secondary and below	459 (17.7)	118 (12.7)		1.5 (1.21 – 1.96), < 0.001
**Household income, n (%)**				
B40	877 (33.8)	324 (34.8)	0.833	Ref
M40	1,121 (43.1)	396 (42.6)		1.1 (0.92 – 1.39), 0.231
T20	600 (23.1)	210 (22.6)		1.1 (0.84 – 1.36), 0.600
**Employment status, n (%)**				
Employed	2,085 (80.3)	708 (76.1)	0.012	Ref
Unemployed	492 (18.9)	208 (22.4)		0.8 (0.69 – 1.04), 0.109
Retired	21 (0.8)	14 (1.5)		0.7 (0.31 – 1.42), 0.290
**Healthcare workers, n (%)**				
No	2,167 (83.4)	839 (90.2)	< 0.001	Ref
Yes	431 (16.6)	91 (9.8)		1.7 (1.34 – 2.26), < 0.001
**Lived with elderly or someone with chronic illness, n (%)**				
No	1,880 (72.4)	696 (74.8)	0.144	-
Yes	718 (27.6)	234 (25.2)		
**History of contact with COVID-19, n (%)**				
No	1,921 (73.9)	747 (80.3)	< 0.001	Ref
Yes	677 (26.1)	183 (19.7)		1.3 (1.07 – 1.61), 0.010
**Had been diagnosed with COVID-19, n (%)**				
No	2,440 (93.9)	857 (92.2)	0.026	Ref
Yes	140 (5.4)	58 (6.2)		0.7 (0.49 – 1.06), 0.099
Prefers not to answer	18 (0.7)	15 (1.6)		0.9 (0.34 – 2.06), 0.705
**Families, relatives, colleagues diagnosed with COVID-19, n (%)**				
No	850 (32.7)	290 (31.2)	0.390	-
Yes	1,748 (67.3)	640 (68.8)		
**Vaccinated for COVID-19, n (%)**				
No	24 (0.9)	106 (11.4)	< 0.001	Ref
Yes	2,571 (99.0)	761 (81.8)		15.4 (9.76 – 24.33), < 0.001
Prefers not to answer	3 (0.1)	63 (6.8)		0.3 (0.08 – 0.93), 0.039
**Children under 12 years, (median, IQR)**				
Number	2, 1 – 2	2, 1 – 3	0.020	1.0 (0.90 – 1.03), 0.276
**Age groups of children, n (%)**				
5 – 11 years	1,970 (75.8)	733 (78.8)	0.065	-
2 – 4 years	1,236 (47.6)	455 (48.9)	0.480	
Below 2 years	425 (16.4)	142 (15.3)	0.437	
**Children had chronic illness, n (%)**				
No	2,483 (95.6)	862 (92.7)	< 0.001	Ref
Yes	99 (3.8)	33 (23.5)		(0.62 – 1.47), 0.826
Prefers not to answer	16 (0.6)	35 (3.8)		0.2 (0.11 – 0.48), < 0.001

^*^p-value of t-test between parents who were willing versus who were not willing or unsure to vaccinate their children against COVID-19

^#^*p*-value of binary logistic regression between parents who were willing versus who were not willing or unsure to vaccinate their children against COVID-19

*SD* Standard deviation, *CI* Confidence interval, *COVID-19* Coronavirus 2019, *B40* Lowest 40% of family income group, *M40* Middle 40% of family income group, *T20* Top 20% of the family income group, *OR* Odds ratio, *IQR* Interquartile range

Sociodemographic factors that influenced the parents’ decision included their age (*P* = 0.046), marital status (*P* = 0.006), region of residence (*P* = 0.001), education level (*P* < 0.001), employment status (*P* = 0.012), and whether they were healthcare workers (*P* < 0.001). Clinical factors that influenced parents’ decision included history of significant contact with COVID-19 (*P* < 0.001), previous diagnosis of COVID-19 (*P* = 0.026), and their COVID-19 vaccination status (*P* < 0.001). Children factors that influenced the parents’ decision included the number of children younger than 12 years (*P* = 0.020) and whether the children had a chronic disease (*P* < 0.001).

Multivariate analysis showed that single parents (odds ratio [OR], 2.0; 95% confidence interval [CI], 1.31–3.04; *P* = 0.001), those with secondary or lower education (OR, 1.5; 95% CI, 1.21–1.96; *P* < 0.001), healthcare workers (OR, 1.7; 95% CI, 1.34–2.26; *P* < 0.001), those who had significant contact with COVID-19 (OR, 1.3; 95% CI, 1.07–1.61; *P* = 0.010), and those who already received COVID-19 vaccine (OR, 15.4; 95% CI, 9.76–24.33; *P* < 0.001) were more willing to vaccinate their children against COVID-19. Conversely, the willingness of parents from the Northern region of the country to vaccinate their children against COVID-19 was significantly lower than those from the Central region (OR, 0.7; 95% CI, 0.53–0.91; *P* = 0.008).

### The reasons parents were willing to vaccinate their children against COVID-19

**Fig. 1:**
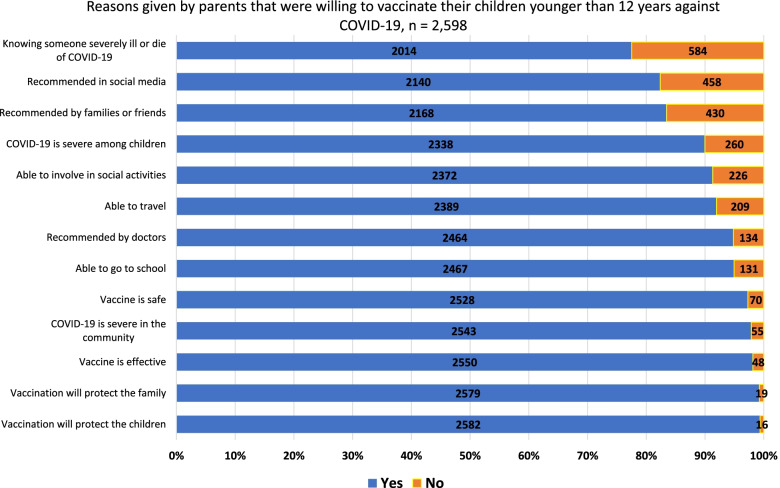
Reasons given by parents that were willing to vaccinate their children younger than 12 years against COVID-19

The majority of the parents agreed with all the reasons provided in the survey (77.5%–99.4%) as the rationale behind their willingness to vaccinate their children against COVID-19 (Fig. 1). The three most common reasons were as follows: 1) the COVID-19 vaccination could protect their children (99.4%), 2) the vaccination of their children could also protect other family members (99.3%), 3) and the vaccine was effective (98.2%). Parents additionally stated that the COVID-19 vaccination in children could contribute to overall herd immunity and children had difficulty adhering to precautionary measures.

### The reasons parents were not willing to vaccinate their children against COVID-19

**Fig. 2:**
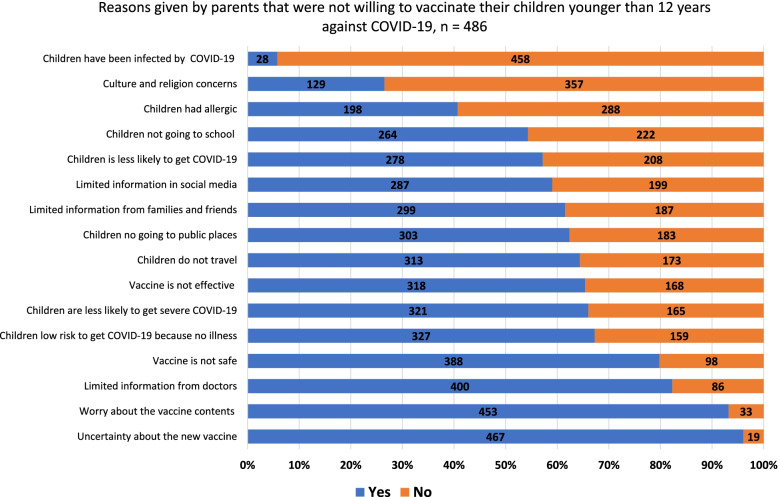
Reasons given by parents that were not willing to vaccinate their children younger than 12 years against COVID-19

The common reasons that were given by parents who were not willing to vaccinate their children against COVID-19 included the uncertainty toward the new COVID-19 vaccine (96.1%), concerns about vaccine contents (93.2%), limited information about the vaccine for children from physicians (82.3%), and the belief that the vaccine was unsafe (79.8%) (Fig. 2). The following reasons were less common among those respondents: a COVID-19 diagnosis in children (5.8%), factors related to culture and religion (26.5%), and allergies (40.7%). Additional reasons that were added by the parents included a bad experience during their COVID-19 vaccination, the belief that an alternative treatment was available to prevent COVID-19 infection, and another belief that children may acquire COVID-19 infection during the vaccination.

### The factors that the parents who were still hesitant would like to consider before allowing their children to be vaccinated against COVID-19

**Fig. 3:**
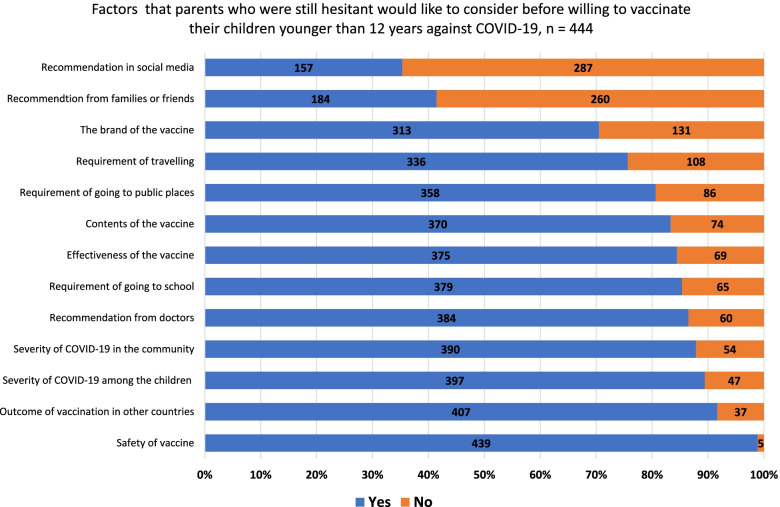
Factors that parents who were still hesitant would like to consider before allowing their children younger than -12 years to be vaccinated against COVID-19

Other than recommendations in social media (35.3%) and recommendations from families or friends (41.4%), the parents would consider all factors (70.5%–98.9%) listed in the survey before allowing their children to get vaccinated against COVID-19 (Fig. 3). No additional concerns were added by the parents.

## Discussion

### General discussion

To date, few studies focused on parents’ willingness to vaccinate children younger than 12 years against COVID-19. Teasdale et al. reported that 49.4% of parents in the United States were willing to vaccinate their children younger than 12 years against COVID-19 [29]. Meanwhile, Almusbah et al. reported that only 25.6% of parents in Arab were willing to do so [30]. Another study by Hetherington et al. reported that 60.4% of mothers in Canada were keen to obtain the COVID-19 vaccine for their children aged 9–12 years [22]. More studies looked at the willingness of parents to immunize their children younger than 18 years against COVID-19. For example, multinational studies by Skjefte et al. and Goldman et al. respectively reported that 69.2% and 65.2% of parents were willing to vaccinate their children in that age group [31, 32]. The willingness of parents to vaccinate children in that age group ranged 44.3%–73.0% in the United States [33–36] and 59.3%–72.7% in China [37, 38], and it was 64.2% in Korea [39], 60.4% in Italy [40], 51.0% in Germany [41], and 48.0% in the United Kingdom [20]. Considerably fewer parents in Turkey (29.0%–36.3%) were willing to vaccinate their children in that age group [23, 42]. In comparison with these studies, parents in our study were more willing to immunize their children (73.6%), even though previous reports showed that parents were more likely to vaccinate their older children, especially teenagers [32, 36]. An explanation of that could be the fact that this study was conducted after the majority of adults in Malaysia had been vaccinated and during a nationwide program to vaccinate teenagers. Goldman et al. reported that caregivers in the United States, Canada, and Israel were more willing to accept the expedited approval of the COVID-19 vaccine for children younger than 12 years after the emergency approval and commencement of the national COVID-19 vaccine program in adults [43].

Based on the available literature, independent predictors of parents’ willingness to vaccinate their children younger than 12 years against COVID-19 included male gender [29, 30], higher educational level [22, 29], higher household income [22, 29], Asian descent the United States [29], more willingness to get the COVID-19 vaccine for themselves [29], and having children with complete routine immunization [22]. In children younger than 18 years, independent predictors of parents’ willingness to vaccinate their children against COVID-19 included the following: male gender [23, 32, 34, 40, 42], older age [31, 34, 40], higher household income [31, 34], willing to receive or already received COVID-19/influenza vaccine [23, 32, 34, 36], perceived threat of COVID-19 [23, 34], healthcare workers [23], and having children with complete routine immunization [31]. Even though the majority of the studies showed parents with higher education were more willing to vaccinate their children [31, 36, 40], Wang et al. [37], Yigit et al. [42], and Temsah et al. reported the opposite findings [44].

In this study, there are several explanations for the factors that significantly affect parents’ willingness to vaccinate their children against COVID-19. First, parents who have already received the COVID-19 vaccine were 15-fold more willing to vaccinate their children, which can be explained by the psychological domain gradient of the Health Belief Model. It states that parents who neither delay nor refuse their vaccination are more likely to vaccinate their children, and vice versa [45]. Furthermore, Bourassa et al. reported health behaviors during the COVID-19 pandemic were heavily influenced by past health behaviors [46], for example, willingness to vaccinate. Second, a higher level of perceived threat particularly among single parents, healthcare workers, and those with a history of significant contact in our study could positively influence willingness to vaccinate children [47]. Third, previous studies have shown that parents with a higher level of education frequently have a higher level of vaccination hesitancy due to safety concerns [48, 49]; therefore, parents with a lower level of education in this study were more likely to vaccinate their children. Fourth, parents from areas with fewer COVID-19 cases and lower vaccination rates, such as the Northern region, were more hesitant to vaccinate their children because they had lower exposure to the infection and, thus, were less concerned about contracting it [50].

Common reasons that were given by the parents that were willing to vaccinate their children younger than 12 years against COVID-19 included the belief that the vaccine can protect their children as well as their family and others, it is effective, and it is recommended by healthcare workers or government [22, 30]. Similar reasons were also given by parents who were willing to vaccinate their children younger than -18 years, with additional reasons—the belief that the vaccine can help control the pandemic and that the benefits of vaccination outweigh the harms [20, 23]. Conversely, uncertainty toward the new vaccine; concerns as to its efficacy, side effect, and safety; and the perception that children were at lower risk for COVID-19 were the main reasons parents hesitated or were not keen to vaccinate their children younger than 12 or 18 years [20, 22, 23, 29, 30]. Parents in our study also reported similar reasons for their willingness or lack thereof to vaccinate their children against COVID-19, except for the outcome of the vaccination program in other countries. The severity of COVID-19 in the community/among children was the main concern among parents who still hesitated. Previous studies demonstrated that acceptance of the vaccine was frequently associated with external factors (such as information about vaccine protection and recommendations of vaccine by healthcare workers or governments), whereas vaccine hesitancy and refusal were mainly due to vaccine-specific factors (such as perceived vaccine safety, efficacy, and disease susceptibility), which might explain the present results [51].

Children consist 28.3% of Malaysian’s population of 32.7 million, and more than half of them were younger than 12 years. Therefore, our finding that a quarter of parents with children younger than 12 years were unwilling or hesitant to vaccinate their children against COVID-19 is worrying. Furthermore, the finding that parents who have not received their COVID-19 vaccine were at a 15-fold higher risk of refusing or hesitating to vaccinate their children against COVID-19 warrants prompt attention. The present study also identified vaccine-specific factors that led to COVID-19 vaccine refusal and hesitancy, as well as external factors that promote a positive attitude toward COVID-19 vaccination. Based on these findings, more targeted health education can be planned to mitigate COVID-19 vaccination refusal and hesitancy in parents of children younger than 12 years.

### Strategies for interventions

First, more health education is needed to increase parents’ awareness of COVID-19 vaccination in children younger than 12 years. Education resources should be comprehensive, multilingual, and layman-friendly to reach out to parents from all walks of life. The common channels that Malaysians use to obtain information on the COVID-19 vaccine, such as electronic media and social media, could serve as an excellent education platform, whereas printed materials and face-to-face public talks may still benefit certain populations, particularly those from rural areas and with a lower level of education [52]. Second, health education should target parents at risk of vaccine refusal or hesitance, such as those yet to receive the COVID-19 vaccine. This group of parents could be reached on social media platforms (such as Facebook and WhatsApp group) that provide inaccurate information. A recent study by Johnson et al. highlighted that there are more antivaccine Facebook pages, they have better network reach, and they are more common among parenting or school groups [53]. MySejahtera, a mobile application developed by the Malaysian government to facilitate COVID-19 contact tracing and vaccination, could assist in identifying unvaccinated parents and subsequently delivering the appropriate information to them. Third, health education should focus on addressing the common reasons for refusing the COVID-19 vaccine, such as uncertainty toward a new vaccine, concerns about its contents and safety, and insufficient information on the topic from physicians. Fourth, health education should highlight the benefits of the COVID-19 vaccine, thus promoting more vaccination rollout. The benefits to focus on include the following: protection of children, family members, and others as well as good efficacy. Fifth, experimental and real-world data comparing health outcomes of children younger than 12 years who received vaccinate compared with those who did not should be provided to parents once available.

### Strengths and limitations

Our study is among the few that assessed parents’ willingness to vaccinate their younger children against COVID-19, and the first such report in Malaysia. This study had a large sample size that was representative of the whole population. The reasons why parents were willing, unwilling, or hesitant to vaccinate their children against COVID-19 were comprehensively evaluated, including open-ended questions for them to express opinions. Nonetheless, this study has several limitations. First, snowball sampling is a nonprobability sampling method. Second, only parents with internet access could participate in this online survey. Third, the number of respondents from the Northern region was relatively low. Fourth, the history of influenza vaccination was not assessed because it was not routinely administered among adults or the younger population in Malaysia. Fifth, parents’ knowledge of COVID-19 and their source of information about COVID-19 was not assessed, which could be a confounding factor.

## Conclusions

Nearly three-quarters of parents in this study were willing to vaccinate their children younger than 12 years against COVID-19. The COVID-19 vaccination history of the parents was the strongest independent predictor for their willingness to vaccinate their children. Perceived efficacy and protection conferred by the COVID-19 vaccine promoted a positive attitude toward vaccination, whereas the uncertainty associated with a new vaccine, concerns about vaccine content and safety, and lack of information from physicians led to vaccine refusal. Therefore, future health education should target parents at risk of vaccine refusal or hesitation, focus to address the common reasons for refusing the COVID-19 vaccine, and highlight the benefits of the COVID-19 vaccine.

## Supplementary Information

**Additional file 1.**

## Data Availability

The datasets generated and/or analysed during the current study are not publicly available due to limitations of ethical approval involving the participants’ data and anonymity but are available from the corresponding author on reasonable request.

## Abbreviations

COVID-19: Coronavirus 2019
WHO: World Health Organization
B40: Lowest 40% of family income group
M40: Middle 40% of family income group
T20: Top 20% of the family income group
SD: Standard deviation
OR: Odds ratio
CI: Confidence internal
