# Supplementary material for: The willingness of parents to vaccinate their children younger than 12 years against COVID-19: a cross-sectional study in Malaysia
Source: BMC Public Health. 2022 Jun 29;22:1265. doi: 10.1186/s12889-022-13682-z (PMC9241237; doi:10.1186/s12889-022-13682-z)
Supplement: Supplementary file 1 — Additional file 1. [file 12889_2022_13682_MOESM1_ESM.docx]

**Supplement Figure 1.**

**Part 1: Socio-demographic characteristics and clinical data of parents:**

1. What is your age?

_______________

1. What is your gender?

|  | Male |
| --- | --- |
|  | Female |

1. What is your marital status?

|  | Married |
| --- | --- |
|  | Single parent (widow/divorced) |

1. What is your highest education level?

|  | Tertiary |
| --- | --- |
|  | Secondary school or below |

1. What is your household income?

|  | B40 (RM6200 or less) |
| --- | --- |
|  | M40 (> RM6200 - 13,000 |
|  | T20 (> RM13,000 |

1. What is your occupation

|  | Employed |
| --- | --- |
|  | Unemployed |
|  | Retired |

1. Are you a healthcare worker?

|  | Yes |
| --- | --- |
|  | No |

1. Where are you currently staying?

|  | Central zone |
| --- | --- |
|  | Southern zone |
|  | Northern zone |
|  | East coast |
|  | Borneo island |

1. Are you staying with elderly or someone with chronic illness?

|  | Yes |
| --- | --- |
|  | No |

1. Do you ever expose to COVID-19?

|  | Yes, required quarantine |
| --- | --- |
|  | Yes, required self-surveillance |
|  | No |

1. Have you been diagnosed with COVID-19?

|  | Yes |
| --- | --- |
|  | No |
|  | Prefer not to answer |

1. Any of your families, friends or colleagues have been diagnosed with COVID- 19?

|  | Yes |
| --- | --- |
|  | No |

1. Have you received your COVID-19 vaccine?

|  | Yes |
| --- | --- |
|  | No |
|  | Prefer not to answer |

**Part 2: Socio-demographic characteristics and clinical data of children:**

1. How many of your children are under 12 years?

_______________

1. Please state down their age:

______________________________________

1. Do any of your children have a chronic illness?

|  | Yes |
| --- | --- |
|  | No |
|  | Prefer not to answer |

**Part 3: Willingness for COVID-19 vaccine:**

1. Are you willing to allow your children under 12 years to receive the COVID-19 vaccine if it is approved by the government later?

|  | Yes |
| --- | --- |
|  | No |
|  | Still unsure |

1. **Questionnaire for parents that willing to vaccinate their children < 12 years old against COVID-19.**

There are various reasons that parents are willing to vaccinate their children < 12 years old against COVID-19. For yourself, what are the reasons that you will allow your children < 12 years old to get the COVID-19 vaccine?

| No: | Reasons: | Answer | |
| --- | --- | --- | --- |
|  |  | Yes | No |
| 1. | COVID-19 vaccine is effective. |  |  |
| 2. | COVID-19 vaccine is safe. |  |  |
| 3. | It is recommended by doctors. |  |  |
| 4. | It is recommended by families or friends. |  |  |
| 5. | It is recommended in social media. |  |  |
| 6. | COVID-19 is severe in the community. |  |  |
| 7. | COVID-19 is severe among children. |  |  |
| 8. | Knowing someone who dies or is severely ill with COVID-19. |  |  |
| 9. | COVID-19 vaccination will protect the children. |  |  |
| 10. | COVID-19 vaccination will protect the family members. |  |  |
| 11. | Children are able to go to school. |  |  |
| 12. | Children able to involve in social activities (such as playing, party, shopping...). |  |  |
| 13. | Children are able to travel. |  |  |
| 14. | Other reasons not mentioned above. | In written: |  |

1. **Questionnaire for parents that are not willing to vaccinate their children < 12 years old against COVID-19.**

There are various reasons that parents are not willing to vaccinate their children < 12 years old against COVID-19. For yourself, what are the reasons that you will not allow your children < 12 years old to get the COVID-19 vaccine?

| No: | Reasons: | Answer | |
| --- | --- | --- | --- |
|  |  | Yes | No |
| 1. | COVID-19 vaccine is not effective. |  |  |
| 2. | COVID-19 vaccine is not safe. |  |  |
| 3. | Worry about the COVID-19 vaccine contents. |  |  |
| 4. | There is uncertainty about the new vaccine. |  |  |
| 5. | Limited information from doctors about the vaccine. |  |  |
| 6. | Limited information from families and friends about the vaccine. |  |  |
| 7. | Limited information about the vaccine in social media. |  |  |
| 8. | Children are less likely to get infected by COVID-19. |  |  |
| 9. | Children are less likely to get the severe disease from COVID-19. |  |  |
| 10. | My children have no illness, therefore low risk to get COVID-19. |  |  |
| 11. | My children have been infected by COVID-19. |  |  |
| 12. | My children have allergic. |  |  |
| 13. | My children not going to school. |  |  |
| 14. | My children not going to public places. |  |  |
| 15. | My children do not travel. |  |  |
| 16. | Culture and religion concerns. |  |  |
| 17. | Other reasons not mentioned above. | In written: |  |

1. **Questionnaire for parents that still hesitate to vaccinate their children < 12 years old against COVID-19.**

There are various reasons that parents still hesitate to vaccinate their children < 12 years old against COVID-19. For yourself, what are the reasons that you will consider before will allow your children < 12 years old to get the COVID-19 vaccine?

| No: | Reasons: | Answer | |
| --- | --- | --- | --- |
|  |  | Yes | No |
| 1. | The effectiveness of the COVID-19 vaccine. |  |  |
| 2. | The safety of COVID-19 vaccine. |  |  |
| 3. | The contents of the COVID-19 vaccine |  |  |
| 4. | The brand of the COVID-19 vaccine. |  |  |
| 5. | The recommendation from doctors. |  |  |
| 6. | The recommendation from families or friends. |  |  |
| 7. | The recommendation in social media |  |  |
| 8. | The severity of COVID-19 in the community. |  |  |
| 9. | The severity of COVID-19 among the children |  |  |
| 10. | The requirement of going to school. |  |  |
| 11. | The requirement of going to public places. |  |  |
| 12. | The requirement of travelling. |  |  |
| 13. | The outcome of vaccination program for children under 12 years in other countries. |  |  |
| 14. | Other reasons not mentioned above. | In written: |  |
